# Low serum melatonin levels are associated with erectile dysfunction

**DOI:** 10.1590/S1677-5538.IBJU.2017.0663

**Published:** 2018

**Authors:** Aliseydi Bozkurt, Mehmet Karabakan, Binhan Kagan Aktas, Murat Gunay, Ercüment Keskin, Erkan Hirik

**Affiliations:** 1 Department of Urology, Erzincan University Mengucek Gazi Research and Training Hospital, Erzincan, Turkey; 2 Department of Urology, Mersin Toros State Hospital, Mersin, Turkey; 3 Department of Urology, Ankara Numune Research and Training Hospital, Ankara, Turkey;; 4 Department of Clinical Biochemistry, Erzincan University Mengucek Gazi Research and Training Hospital, Erzincan, Turkey

**Keywords:** Erectile Dysfunction, etiology [Subheading], Melatonin, Oxidative Stress

## Abstract

**Objective:**

Melatonin is a hormone secreted from the pineal gland and has anti-oxidative and anti-inflammatory effects. Oxidative stress is considered as an important factor in the etiology of erectile dysfunction (ED), and in many experimental models, positive results have been obtained with melatonin treatment. This study aimed to measure serum melatonin levels in ED patients and to investigate the possible relationship between ED and melatonin levels.

**Materials and Methods:**

Sixty-two patients diagnosed with mild, moderate or severe ED according to the five-item International Erectile Function Index (IIEF-5) and 22 healthy individuals were included in the study. The serum melatonin levels, anthropometric data, and other biochemical and hormonal parameters of all the subjects were recorded. Detailed anamnesis was also obtained in terms of diabetes, hypertension, cardiovascular diseases, smoking status, and alcohol use.

**Results:**

The serum melatonin level was found 34.2±13.3 ng/dL in the mild ED group, 33.3±14.7 ng/dL in the moderate ED group, 34.8±17.2 ng/dL in the severe ED group, and 44.6±16.5 ng/dL in the control group. The serum melatonin levels were significantly lower in all ED groups compared to the control group (p=0.019). There was no significant difference in the serum melatonin levels between the three ED groups. Diabetes, hypertension, cardiovascular diseases, smoking and alcohol use were not significantly different between the ED groups (p>0.05).

**Conclusion:**

We consider that if our findings are supported by further studies with larger populations, the measurement of the serum melatonin level may have a future role in the diagnosis and treatment of ED.

## INTRODUCTION

Erectile dysfunction (ED) is defined as an inadequate or unsustainable penile erection for satisfactory sexual performance (1). The incidence of ED has been reported to increase with age, reaching 20-40% in males in the age range of 60-69 years and 50-100% in those aged 70 to 80 (2). In ED etiology, among the major causes are chronic diseases, such as hypertension, diabetes mellitus, and coronary artery diseases (CAD), as well as the negative effects of the drugs used for these conditions (3). The mechanism underlying these etiologic diseases is the development of degenerative changes leading to endothelial dysfunction (4). The pathophysiological mechanism of endothelial dysfunction is multifactorial and often characterized by degraded nitric oxide bioavailability, decreased vasodilatation, and worsening inflammation, preceded by atherosclerotic lesions (5). Nitric oxide (NO) production plays a central physiological role in the erection process. Neurogenic nitric oxide (NO) is considered as the most important factor for the relaxation of corpora cavernosa (CC) and the penile vessels necessary for erectile response (5).

Melatonin is a hormone secreted from the pineal gland and has anti-oxidative and anti-inflammatory effects (6). Studies have shown that melatonin detoxifies highly reactive hydroxyls (OH) *in vitro* and sweeps free radicals (7). Although melatonin has been reported to increase all aspects of sexual activity in rats (8), it has also been stated that chronic administration of this hormone may lead to the inhibition of sexual activity of male rats (9). Previous research suggests that melatonin may have positive effects on erectile function. It was also demonstrated that acute administration of melatonin restored complete sexual activity in selected impotent male rats (10). Furthermore, melatonin treatment reduced oxidative stress and improved the contractility of CC in diabetic Wistar rats (11). Melatonin treatment has also been reported to result in reduced / prevent the functional and morphological changes induced by chronic ischemia in penile structure and function (12).

Erection is essentially a neurovascular event that requires intact and functional endothelium and smooth muscle in the corpus cavernosum (CC) (13). It is known that atherosclerosis is an inflammatory process involving a number of proinflammatory cytokines, representing an increased state of oxidative stress (14). Melatonin protects tissues from oxidative damage induced by the various processes that produce free radicals (15). In addition, melatonin acts an indirect antioxidant by activating major anti-oxidant enzymes, such as superoxide dismutase (16). Evidence in the literature suggests that melatonin has a role in reducing oxidative stress induced in many organs by diabetes (17, 18).

However, to our knowledge, no studies have explored the relationship between serum melatonin levels and ED; therefore, we decided to investigate the possibility of melatonin deficiency in ED patients to serve as a potential simple to use diagnostic marker.

## MATERIALS AND METHODS

Prior to the commencement of the study, approval from the local ethics committee and the informed consent of all the participants were obtained. A total of 62 ED patients and 22 healthy volunteers (control) were included in the study. The patients referred to the urology clinic were divided into three groups as having mild, moderate and severe ED.

All the patients had ED complaints about their sexual activities for at least six months. The erectile function of all the patients was determined according to the five-item version of the International Index of Erectile Function (IIEF-5) (19). Based on their IIEF-5 scores, participants were divided into three ED groups as follows: severe (score: 1-7), moderate (8–16), and mild (17–21). All the participants had been sexually active for the previous six months and responded to the IIEF-5 items concerning sexual activities.

The exclusion criteria were: the presence of neurological disorders, history of pelvic trauma, anemia, major psychiatric diseases, psychogenic erectile dysfunction, thyroid disease, acute or chronic urinary tract disease and end stage renal disease; having used medication that would affect their sex hormones or vitamin metabolism within the last three months; and being under treatment for ED. The informed consent of the participants was taken prior to the collection of blood samples.

### Medical History and Physical Examination

Demographic and medical information was obtained from all the participants, including age, medical history, presence of hypertension, smoking status, duration of sexual dysfunction. Physical examination comprised the digital rectal examination (DRE), genital examination and measurement of height and weight and calculation of body mass index (BMI) by dividing weight in kilograms by height in meters squared. The patients suspected having prostate cancer in DRE or symptoms of hypogonadism, were excluded from the study.

### Laboratory tests

For the laboratory tests, blood samples were taken from all the participants at 8 a.m. after fasting for 12 hours and stored at -20° C until assayed. Fasting blood glucose (FBG), total testosterone, triglyceride, low-density and high-density lipoprotein cholesterol (LDL-C and HDL-C), and serum melatonin levels were recorded. Serum melatonin level was measured using an enzyme-linked immunosorbent assay (SunRedbio ELISA Kit) and an Epoch microplate spectrophotometer (BioTek Instruments, Inc., Winooski, VT, USA). Serum testosterone level was measured using an enzyme-linked immunosorbent assay (Siemens Centaur XP Kit, Germany).

### Statistical analysis

For discrete and continuous variables, descriptive statistics (mean, standard deviation, n and percentile) were given. To compare the differences between three and more groups, one-way analysis of variance was used when the parametric test prerequisites were fulfilled, and the Kruskal Wallis test was used when such prerequisites were not fulfilled. The relationship between two continuous variables was assessed by the Pearson Correlation Coefficient, and by the Spearman Correlation Coefficient when the parametric test prerequisites were not met. Data (SPSS, Chicago IL, Version 17) was assessed in the SPSS package program (Chicago IL, Version 17). p<0.05 was taken as significance levels.

## RESULTS

The mean ages of the participants were 54.7±7.6, 54.8±9.5 and 57.0±7.1 in the mild, moderate and severe ED groups, respectively, and 51.9±7.6 in the control group. There was no statistically significant difference between the four groups in terms of the mean age, BMI, HT, smoking, alcohol use, diabetes mellitus, total testosterone, LDL-C, HDL-C, triglyceride and cholesterol (p>0.05). However, statistically significant differences were obtained between the groups concerning melatonin and FBG (p<0.05). The mean serum FBG level in severe ED group was significantly highest in all groups, (P<0.05). and FBG level were 103 mg/dL, 115.6 mg/dL and 127 mg/dL in the mild, moderate and severe ED groups, respectively, and 100 mg/dL in the control group. The mean IIEF-5 scores were 5.85±0.93, 10.22±0.8, 15.26±3.2 and 24±1.38 in the severe, moderate, mild ED and control groups, respectively. Furthermore, all patients in the ED groups had a lower IIEF-5 score than the control group (p<0.05). This shows that the highest total testosterone level was observed in the control group, but the difference between the other groups wasn't statistically significant.

Table-1 presents clinical information and the fasting endocrine values of all participants. The serum melatonin levels were significantly lower in all ED groups compared to the control group (p=0.019): 34.2±13.3 ng/dL in the mild ED group, 33.3±14.7 ng/dL in the moderate ED group, 34.8±17.2 ng/dL in the severe ED group, and 44.6±16.5 ng/dL in the control group. There was no significant difference among the ED groups in terms of melatonin levels (p>0.05). In all ED groups, there was a statistically significant association (r=0.586, p=0.047) between IIEF-5 and melatonin levels with the increase being parallel in both parameters.

**Table 1:** participant clinical data and fasting endocrine values.

	Severe ED (n:20)	Moderate ED (n:23)	Mild ED (n:19)	Control (n:22)	*p*
Clinical values					
Age (year)	57.05±7.1	54.8±9.5	54.7±7.6	51.9±7.6	0.23
IIEF-5 score	5.85±0.93	10.22±0.80	15.26±3.21	24 ±1.38	0.001**
BMI, kg/m^2^	30.2±3.0	28.8±4.2	29.6±3.8	27.5±2.9	0.513
Hypertension (%)	20.0	8.7	10.5	9.1	0.704
Smoking (%)	60.0	47.8	30.0	26.3	0.082
Diabetes (%)	15.0	8.7	5.3	9.1	0.800
CAD (%)	15.0	8.7	10	5.5	0.109
Haematochemical values					
FBG (mg/dL)	127 (73-277)	115.65 (72-209)	103 (81-160)	100 (86-162)	0.036*
TT (ng dL^−1^)	380.7±53.5	306.17±83.8	404.6±143.4	341.0±106.7	0.46
LDL (mg/dL)	127.1±37.0	109.5± 30.1	107.5±33.4	110.6±40.8	0.264
HDL (mg/dL)	41.80±9.2	42.2±8.3	42.1±10.4	44.3±7.12	0.196
TG (mg/dL)	209.5±100.6	187.8±97.0	150.6±61.6	176.5±83.2	0.231
Total cholesterol, (mg/dL)	195.6± 48.9	172.7±28.1	205.8± 33.3	182.0± 36.8	0,28
Melatonin(ng/dL)	34.8±17.2	33.3±14.7	34.2±13.3	44.6±16.5	0.019*

**IEEF-5 score** = International Index of Erectile Function 5; **BMI** = body mass index; **CAD** = Coronary artery disease; **FBG** =Fasting blood glucose; **LDL** = Low-density lipoprotein; **HDL** = High-density lipoprotein; **TG** = Triglyceride; **TT** = Total Testosterone

* p<0.05;

** p<0.01

## DISCUSSION

We found that in the present study, serum melatonin levels in ED patients were found to be significantly lower than controls. In their study, Qiu et al. detected increases in both erectile function and the endothelial density of the CC in diabetic rats after melatonin treatment (20). In another study conducted on diabetic rats, melatonin was reported as contributing to many histological and functional changes through its local anti-oxidative effect on CC and having a relaxing effect on smooth muscles in CC (11). Drago et al. (10) demonstrated that selected impotent male rats regained full sexual activity following a low dose of melatonin treatment (10-100 mg/kg). Similarly, Paskaloglu et al. (11) reported reduced oxidative stress and improved CC contraction in diabetic Wistar rats following melatonin treatment (6 mg/kg per day for six weeks). In a recent animal study, Sawada et al. (12) reported that after melatonin treatment (20 mg/kg per day for 8 weeks), erectile responses were regained, collagen deposition in CC was reduced, contractile and relaxant responses were protected in isolated CC strips, and there was increased neuronal and enothelial NO synthase (NOS) and decreased inducible NOS expression in CC. It is considered that ischemic erectile tissue dysfunction may involve multiple mechanisms, such as chronic nutrient deficiency, hypoxia and metabolic wastes, and may affect NO production or function due to cytotoxicity (21). Sawada et al. (12) also showed that the down-regulation of eNOS and nNOS expression might lead to a further deterioration of corporal tissue and eventual ED. In addition to that, eNOS and nNOS proteins were found to be reduced tissues of chronic ischemic CC, and both of which were significantly improved by melatonin treatment. As a result of chronic use, melatonin exhibits protective effects on chronic ischemic CC by scavenging free radicals and through anti-oxidative properties.

Endothelial dysfunction leading to atherosclerosis and/or smooth muscle dysfunction plays a major role in the pathogenesis of both ED and CAD.

The results of the study by Tavukcu et al. (22) indicated the critical pathogenic contribution of increased oxidative stress to ED caused by spinal cord injury and they finally suggested that melatonin and tadalafil combination leaded to similar beneficial effects through different mechanisms of action.

The circadian organisation of melatonin on human physiological functions such as immune system, antioxidant defences, haemostasis and glucose regulation was demonstrated (23). Javanmard et al. (24) reported that even in patients with severe and advanced atherosclerotic plaques, melatonin may have beneficial effects on endothelial dysfunction. In that study, the authors found a significant reduction in the mean levels of inter-cellular adhesion molecule, vascular cell adhesion molecule and C- Reactive Protein following one month of melatonin treatment. Furthermore, they noted higher serum NO levels in the study group compared with the controls. The authors concluded that melatonin might reduce the markers of endothelial cell damage and increase vasodilator cytokines. These positive results obtained with the replacement of melatonin deficiency made us think that melatonin deficiency may be of importance in ED etiology. And we think that melatonin treatment may be useful for both atherosclerosis and ED.

In human studies, reduced levels of melatonin were reported to exist in patients with type 2 diabetes (25) and hypertension (26). It has also been shown that blood melatonin levels were correlated with the severity of disease in patients with cardiovascular disease (27). These studies show that melatonin deficiency might compromise many systems and it could play a causative role for many disease. In the present study, serum melatonin levels in ED patients were found to be significantly lower than controls. However, no significant difference was detected among the ED groups in melatonin levels. There was not any proportional relationship between melatonin deficiency and ED severity in our small sample. This result made us think that the level of melatonin deficiency may not reflect the ED severity.

As far as we know, this is the first study evaluating serum melatonin level as a causative factor in this patient group. A low serum melatonin level may result in an inadequate erection by preventing sufficient antioxidant capacity. There is a need for additional studies to determine the exact role of melatonin deficiency in ED patients. The limitations of our study were the absence of Doppler ultrasound findings, the lack of a treatment group and follow-up data on melatonin levels and the small sample size. Future studies may asses the association or a possible correlation between serum melatonin levels and Doppler ultrasound parameters of erectile function. Furthermore, serum inflammatory markers may also be measured for investigating the relationship between melatonin and inflammation level.

## CONCLUSIONS

In the present study, we found a significant relationship between ED presence and low serum melatonin levels. This relationship suggests that melatonin deficiency may be of importance in ED etiology. If larger clinical trials confirm our findings, measurement of serum melatonin level may have an additive future role in ED diagnosis and melatonin replacement would find an indication for ED treatment.
